# Adaptive Immunity and Inflammation

**DOI:** 10.1155/2015/575406

**Published:** 2015-02-22

**Authors:** Brancaleone Vincenzo, Iqbal J. Asif, Paschalidis Nikolaos, Maione Francesco

**Affiliations:** ^1^Department of Science, University of Basilicata, Via Ateneo Lucano, 85100 Potenza, Italy; ^2^Sir William Dunn School of Pathology, University of Oxford, South Parks Road, Oxford OX1 3RE, UK; ^3^Cellular Immunology, Biomedical Research Foundation Academy of Athens, 11527 Athens, Greece; ^4^Department of Pharmacy, University of Naples Federico II, Via Domenico Montesano 49, 80131 Naples, Italy

Inflammation is part of a complex biological response to injury as a result of different stimuli such as pathogens, damaged cells, or irritants. Local signals at the sites of inflammation mediate rapid cells mobilization and recruitment and dictate differentiation programs whereby these cells drive clearance of “inflammatory inducers” and promote resolution and restoration of tissue homeostasis. However, persistent inflammatory stimuli or dysregulation of mechanisms of the resolution phase can lead to chronic inflammation. This phenomenon, from a “temporal point of view,” distinguishes a first cellular subset that responds to proinflammatory stimuli, commonly referred to as innate immunity (PMN, monocytes), later followed by a second phase, classically catalogued as the adaptive immune response (T and B lymphocytes). Inflammation is more generally associated with the innate immune response, however, increasing experimental and clinical evidence has highlighted its importance in antigen driven adaptive immune responses.

Intriguingly, the hypothesis that components of adaptive immunity involve the generation of memory cells which can also fuel the chronic nature of inflammation driven by the adaptive arm of the immune response is now emerging. This “novel view” supports the view that lymphocytes cooperate with innate immune cells and collectively orchestrate the inflammatory response.

Original research and review articles successfully submitted to this special issue have stimulated the continuing efforts to understand the interaction between adaptive immunity and the inflammatory process. This special issue, also pays particular attention to the role of proinflammatory Th17 T cells and T cell derived cytokines in autoimmune, cardiovascular, and infectious diseases (“Interleukin-17A Exacerbates Ferric Chloride-Induced Arterial Thrombosis in Rat Carotid Artery,” authored by F. Maione et al.; “Th17 Cells in Autoimmune and Infectious Diseases,” authored by J. F. Zambrano-Zaragoza et al.; “Limited Applicability of GW9662 to Elucidate PPAR*γ*-Mediated Fatty Acid Effects in Primary Human T-Helper Cells,” authored by A. Jaudszus et al.). Moreover, this special issue has highlighted the importance of lymphocytes involvement during acute and chronic inflammation (“Live Combined* Bacillus subtilis *and* Enterococcus faecium *Ameliorate Murine Experimental Colitis by Immunosuppression,” authored by S. Chen et al.; “Contradictory Immune Response in Post Liver Transplantation Hepatitis B and C,” authored by A. Takaki et al.; “Neurotensin Decreases the Proinflammatory Status of Human Skin Fibroblasts and Increases Epidermal Growth Factor Expression,” authored by L. P. da Silva et al.).

We hope that this special issue will stimulate the interest of scientists working in different areas of inflammation and immunology and highlight the need for continuing research in the area.

*Brancaleone  Vincenzo* *Brancaleone  Vincenzo* *Iqbal  J.  Asif* *Iqbal  J.  Asif* *Paschalidis  Nikolaos* *Paschalidis  Nikolaos* *Maione  Francesco* *Maione  Francesco*

